# Empowerment in structures. Practical-ethical considerations of the preconditions for technology-assisted dementia care in Germany based on an expert-interview study

**DOI:** 10.3389/fpsyt.2024.1437967

**Published:** 2025-01-10

**Authors:** Johannes Welsch, Silke Schicktanz

**Affiliations:** Department of Medical Ethics and History of Medicine, University Medical Center Göttingen, Göttingen, Germany

**Keywords:** empowerment, dementia, intelligent assistive technology, digitalization, exogenous preconditions, policy recommendations

## Abstract

**Background:**

Intelligent assistive technologies (IAT) have become more common in dementia care. Ethical reflection on technology-assisted dementia care (TADC) has focused so far mainly on individual and interpersonal implications (e.g., self-determination, (in)dependence, safety or privacy issues, caregivers’ support and cost-efficiency). From an empowerment-sensitive perspective, however, the societal, political, economic and technological preconditions for TADC should be more deeply analyzed in terms of their accelerating or inhibiting effects on technology development, implementation and usage. Therefore, the aim of this study was to explore these preconditions in the German context and so to contribute to more empowerment-sensitive structures.

**Methods:**

Semi-structured interviews were conducted with 20 German-speaking experts from health care, health policy and the fields contributing to IAT (e.g., computer science, engineering). Thematic content analysis was used to analyze the data.

**Findings:**

The experts’ assessments of the current preconditions for TADC in Germany were starkly ambivalent. In the field of „society”, they identified digitalization, a change in mentality towards IAT and demographic change as accelerators, unequally distributed digital literacy, misleading perceptions and a lack of affinity as inhibitors. In the field “politics - regulation - economy”, experts identified scarcity of public resources, growing private wealth and regulatory progress as accelerators and unclear financing options, an uncertain market, data protection and ethical challenges as inhibitors. In the field “technology”, they identified progress in basic technical research and improved customizability and interconnectivity as accelerators, while deficient digital infrastructure, a lack of user participation, dementia-specific challenges and challenges regarding data collection and security were seen as inhibitors.

**Conclusions:**

TADC promises an empowerment of persons with dementia, e.g. by enhancing their self-determination, increasing their independence from social control and by allowing more social participation. Yet its societal, political, economic and technological environments preconfigure the likelihood of successful empowerment as a socio-technical practice within TADC. Accelerators in the fields of society, politics-regulation-economy and technology need to be consolidated and strengthened. Inhibitors need to be mitigated, e.g. by with new educational, political and market economic policies. We make policy recommendations based on these conclusions.

## 1 Introduction

In recent decades, the normative concept of good ageing has changed fundamentally. Today, it focuses on sustained health and productivity, independence, self-determination and social participation for as long as possible. Good ageing means realizing these normative goals even if one is in need of care, for example due to late onset dementia. Moreover, since care is recognized as a continuum starting with informal care by relatives at home and extending to highly professionalized care in care facilities, this ideal holds in all phases of care (1–4).

Alongside to this normative change, care for ageing persons and persons with dementia is subject to ever more intensive technological development. As a result of advances in computer science and engineering, intelligent assistive technologies (IAT) are being used increasingly to maintain or improve individual functioning. “Intelligent” means that these technologies analyze their environment using sensors and (different forms of) artificial intelligence (AI) and, consequently, operate in a somewhat autonomous manner (5, 6). Examples of such IAT include smart GPS tacking systems that can learn the usual routes of their users and report deviations, smart home systems that use sensors to detect falls and call for help, and (humanoid) care robots that serve as interaction partners for their users (7).

In response to the typical symptoms of dementia — memory impairment, impairment of executive functions, attention, social skills and judgment abilities —, IAT are being implemented with increasing regularity and intensity in dementia care. Its use is intended to increase the safety and, thereby, independence of persons with dementia, to enable them to remain in their own homes longer and to participate more actively in social life (4, 7–9). Additionally, IAT is intended to relieve the physical and psychological burden of family and professional caregivers, to mitigate the shortage of skilled nursing staff and to increase the overall quality of dementia care (4).

Due to these normative goals, ethical reflection on technology-assisted dementia care (TADC) has so far focused primarily on individual and interpersonal implications and on the participation of users in technology development (10–14). With this focus, however, other crucial aspects of TADC are fading from view: TADC is a socio-technical practice of empowerment. As such, it is accelerated and inhibited by its social, political, economic and technological preconditions. Thus, a serious analysis of the actual potential for TADC to promote interpersonal empowerment means highlighting the significant impact of these exogenous preconditions for its likelihood of success.

By empowerment, we mean, first, the endeavor to reduce dependencies in asymmetrical interpersonal, social and political relationships and to support individuals’ power of self-determination. Originally, its focus was solely on pre-existing power relations, i.e. on social and political structures that affect or limit the possibility of individuals and groups to practice self-determination, to be independent and to participate in social and political life. This includes social and political participation of members of marginalized groups (15–17). Secondly, we mean thereby the sum of different social and socio-technical practices: social practices utilizing technology with which these goals are pursued. Originally, empowerment started in community psychology, emancipatory pedagogy and social work, and addressed primarily marginalized socio-economic groups. However, in recent decades, it has become increasingly important in healthcare because of the need to transform asymmetric power-relations such as those between patients and professionals (17–19). Such goals formulated in the empowerment concept for health contexts are particularly relevant for areas of chronic illnesses and in long-term care (17). In a participatory study conducted by McConnell et al (20), people with dementia (PWD) defined empowerment as following: a “confidence building process whereby PWD are respected, have a voice and are heard, are involved in making decisions about their lives and have the opportunity to create change through access to appropriate resources.” In addition to this social practice of empowerment, IAT can be utilized to increase the independence, social participation and, hence, the self-determination of people with chronic conditions. Thereby, empowerment should be discussed nowadays as a socio-technical practice which utilizes technologies. In conclusion, the concept of empowerment provides more reflective potentiality than the traditional ethical principle of autonomy. It can be adapted in a particularly suitable way for a structure-sensitive reflection of socio-technical practices and their exogenous preconditions in society, politics, economy and technology development. Such preconditions preconfigure the possibility of fair access to TADC.

Against this backdrop, this study explores current preconditions for the development, implementation and usage of IAT in dementia care in Germany and discuss them from an empowerment-ethical perspective. A further aim is to contribute to more empowerment-sensitive structures by providing concrete policy recommendations.

To this end, we conducted a qualitative interview study with German experts to learn more about the structural preconditions and the opportunities and risks linked to TADC. In the following, we present the results of the expert study that relate to the preconditions for TADC. Findings on the opportunities and risks of TADC for people with dementia, their relatives and professional caregivers, as well as the care system, have been already published (21). In the discussion, we focus on four identified preconditions —digitalization, unequally distributed digital literacy, deficient digital infrastructure and unclear financing options— and their impact on TADC as a socio-technical practice of empowerment. We conclude with policy recommendations addressing relevant stakeholders in TADC.

## 2 Methods

We conducted a qualitative interview study with German-speaking experts. This design was particularly useful as the question of this study has not yet been adequately addressed in the scientific literature. Furthermore, the experts included have privileged access to the knowledge and debates of their professions and also can potentially influence public and political debates revolving around the preconditions investigated here.

### 2.1 Sample definition and participants

To be included in the study, participants had to belong to one of the following three groups (22, 23):

1. experts in the field of technology research or development related to IAT and/or people with dementia; or.
2. experts in a health care field such as health care policy, health care administration, long-term care insurance or patient organizations; or.
3. experts in the field of nursing profession policy.

Participants were identified based on their professional background as stated in job descriptions and academic profiles, their expertise in the field as evidenced by publications and professional activities and targeted internet research (Google and Google Scholar). Suitable experts were then invited by email to participate in the study. The experts contacted initially were asked to recommend other experts whom they considered relevant to the study. All invitation emails included a description of the research project, a data protection declaration and a declaration of consent for signing.

Of the 26 experts invited to participate in the study, 20 from a broad spectrum of relevant disciplines participated (**Table 1**). Three individuals did not react at all, two declined participation due to time constraints and one declined due to self-assessed lack of expertise.

**Table 1:** Groups of experts and related participants with professional background.

Group	Participants and professional background
1. Technology research and development	1. Expert 2, Engineer 2. Expert 6, Development of IAT 3. Expert 7, Computer Scientist 4. Expert 13, Computer Scientist 5. Expert 14, Engineer 6. Expert 16, Computer Scientist 7. Expert 18, Development of IAT 8. Expert 19, Computer Scientist 9. Expert 20, Engineer
2. Healthcare	1. Expert 1, Representative of a patient organization 2. Expert 5, Representative of a welfare agency 3. Expert 8, Representative of an association of private nursing care providers 4. Expert 9, Representative of federal healthcare politics 5. Expert 11, Representative of a welfare agency 6. Expert 12, Representative of a public long term care insurance 7. Expert 10, Representative of research funding 8. Expert 15, Representative of a private long term care insurance 9. Expert 17, Representative of the healthcare administration of a federal state
3. Nursing profession policy	1. Expert 3, Nursing Management Executive 2. Expert 4, Member of a Nursing Chamber

### 2.2 Interview guide

Following the methods of qualitative research (24), we developed a semi-structured interview guide. The interview guide comprised 15 questions designed to elicit experts’ perceptions and assessments of (1) the preconditions for the development and use of IAT in dementia care, (2) the opportunities and risks of using IAT for people with dementia, family and professional caregivers and (3) the technical and ethical criteria of good IAT in dementia care.

The guide was pre-tested with one participant to check the comprehensibility and factual appropriateness of the items. Following the pre-test, we made minor wording corrections and summarized the questions about opportunities and risks for the different user groups.

### 2.3 Procedure: interview setting and recording

The interviews were conducted from July 2020 to March 2021. After 20 interviews, thematic saturation was observed so no further attempts were undertaken to recruit more experts (cf. 25). Sixteen of the interviews were conducted using videoconferencing systems (Zoom, Microsoft Teams); three interviews were conducted by telephone; one interview was conducted face-to-face. The reason for all but one of the media-mediated interviews was COVID19-induced limitations, particularly social distancing and travel restrictions.

The interviews conducted online were recorded using the recording instruments of the videoconferencing systems and stored on an on-duty hard drive. The telephone and in-person interviews were recorded using a voice recorder. Subsequently, the audio recordings were transcribed verbatim in German; four interviews were transcribed by Johannes Welsch (anonymized for review), the rest by an external service provider who signed a confidentiality agreement.

### 2.4 Interview analysis

The analysis was conducted using the methods of Qualitative Content Analysis (26). For the purpose of qualitative content analysis, a German-language coding guide was developed by Johannes Welsch. To this end, the researchers familiarized themselves with the transcribed interviews by reading them several times and writing memos. In a second step, a preliminary coding guide was drafted with main categories corresponding to the items contained in the interview guide. The coding guide then included code names, rules for coding and anchor quotes. To ensure intercoder reliability, five transcribed and anonymized interviews were independently coded by Johannes Welsch) and the EIDEC project research assistant, Sabrina Krohm. After minor adjustments regarding the coding rules, intercoder reliability was established.

In the fourth step, all material was coded on the basis of the main categories. In the fifth step, all text passages coded with the same main category were compiled. In the sixth step, subcategories were formed for the respective main categories from the material thus compiled and structured. The complete material was then coded in the seventh step using the differentiated coding guide. In the eighth step, a final analysis of the data was carried out with the selection of anchor quotes.

The interview guide had defined three main themes for the qualitative analysis of the interviews: (1) preconditions for the development and use of IAT in dementia care; (2) opportunities and risks of the use of IAT in dementia care for affected persons, family and professional caregivers; (3) criteria of good IAT for dementia care. The preliminary analysis revealed that main theme 1 in particular is interesting from an ethical point of view as it reveals often-overseen structural aspects of technology-assisted dementia care. Main theme 2 covers the broader spectrum of empowerment-ethical implications of the implementation and usage of IAT in dementia care for individuals and interpersonal relations (self-determination, independence and social participation). With regard to the topic of this paper, we will focus on the main theme 1 in the following.

### 2.5 Translation

For the purpose of publication, the quotes from interviews were translated from German into English. Earlier, also the codes were translated from German into English for a cross-cultural publication on the opportunities and risks of TADC.

## 3 Results

The qualitative content analysis revealed that the interviewees mentioned both accelerating and inhibiting preconditions for the development, implementation and use of IAT in dementia care in response to the questions of main theme 1. We identified a total of eleven accelerating and thirteen inhibiting factors (**Table 2**). In the process of the qualitative analysis, we decided that these preconditions can be subsumed under three exogenous structural fields: (1) society, (2) politics - regulation - economy and (3) technology.

**Table 2:** Accelerating and inhibiting preconditions for the development, implementation and usage of IAT in dementia care.

Exogenous structural field	Accelerators	Inhibitors
Society	1. Digitalization 2. Change in mentality 3. Demographic change 4. Shortage of skilled nursing staff 5. COVID19 pandemic	1. Unequally distributed digital literacy 2. Lack of affinity for technology/resentments 3. Misleading technology perception
Politics - Regulation - Economy	6. Growing private wealth 7. Scarcity of public resources 8. Enhanced funding policy 9. Regulative advances	4. Unclear financing options 5. High business risks 6. Uncertain market 7. Challenges regarding data protection and ethics 8. Short research funding periods
Technology	10. Advances in basic technical research 11. Enhanced customizability and interconnectivity	9. Deficient digital infrastructure 10. Lack of potential users’ participation 11. Dementia-specific challenges in development and implementation 12. Focus on innovation 13. Challenges regarding data collection and security

Results are presented below, structured by these three fields. A presentation of accelerators and inhibitors in each field shows ambivalence of TADC preconditions.

### 3.1 Society: digitalization without digital literacy?

In the exogenous structure field “society,” we derived categories of societal developments and transformation procedures such as *digitalization*, the *COVID19 pandemic*, a *change in mentality* among the older generation regarding new technologies as well as need for care, *demographic change* and the *shortage of skilled nursing staff.* All of these were identified as societal preconditions accelerating the development and usage of IAT in dementia care. As societal preconditions, they are driven by explicit and often implicit socio-cultural developments that are not clearly governed politically or economically.

According to the experts, the most important precondition in this field is the digitalization, i.e. the digital transformation of (nearly) all individual and societal practices and realities. Various developments and factors mentioned by the experts can be subsumed under this term. For example, a participant from a welfare agency identified the “[ … ] *increasingly natural use of technology in everyday life* [ … ]” as an accelerating factor (Expert 11). A health-system administrator recognized technology as an increasingly “[ … ] *fundamental part of the reality of life* [ … ]” of most people (Expert 17). Overall, the affinity for technology is increasing in everyday life (Expert 5). Thus, the desire, especially among younger nurses, to use digital technologies in their professional practice is growing (Expert 9; Expert 8; Expert 1). Regarding the opportunities associated with technology use by people with cognitive and/or physical disabilities, a technology researcher emphasized:

“Well, I guess we all use technology and benefit from it and see it as another opportunity for interaction. So there is no reason at all why people with impairments should not also benefit from digital technologies.” (Expert 2).

Some experts from groups 2 and 3 noted that digitalization has been accelerated additionally by the *COVID19 pandemic*. The pandemic has triggered a “*boost*” (Expert 12; Expert 15) in overall technology use and in financial flexibility on the side of policymakers (Expert 17; Expert 3; Expert 9).

Another accelerating precondition was found to be a *change in mentality* among the older generation: Ageing people and those in need for care today have precise ideas about what their life in old age should look like (Expert 9). Here, the desire to remain in one’s own home for as long as possible was identified as central (Expert 5; Expert 9; Expert 10; Expert 17; Expert 18).

Furthermore, the experts named the *demographic change* (ageing of the population) as an accelerating factor for the development and use of IAT in dementia care (Expert 1; Expert 7; Expert 14). This was linked to the ongoing *shortage of skilled nursing staff* and the *decreasing number of informal caregivers* (Expert 7).

According to the interviewed experts, these accelerating preconditions are counterbalanced by inhibiting factors in society. The experts identified a *lack of or unequally distributed digital literacy*, *unclear terms* and associated *misleading perceptions of technology* as well as a *lack of affinity for technology and resentments*, particularly among professional nurses, as inhibiting preconditions.

As a decisive inhibitor the experts identified the *lack of or unequally distributed digital literacy*. An expert stated:

“There is very little knowledge about technology in the normal population.” (Expert 14).

Another interviewee problematized the unequal distribution of the degree of digital literacy necessary for creative technology usage:

“Of course, you need competencies. And these are very unequally distributed. There are people who happen to have a relative who is very tech-savvy and then it works, the relative shares his knowledge. And then there are others who just do not have such a relative and are then cut off from possibilities and opportunities.” (Expert 2).

In addition, the challenge of lacking digital literacy and gaining competencies was mentioned with regard to professional caregivers:

“Well, there are 1.2 million people working as professional nurses. Very few of them have profound digital literacy” (Expert 20).

Moreover, they simply lack the time to acquire these competencies (Expert 4).

Additionally, unclear terms and misleading perceptions of technology were identified as inhibiting factors in the field of society. A federal health care policymaker stated:

“Many people do not know what is meant, but we still use simplifying buzzwords like robotics or digitalization [ … ].” (Expert 9).

A third inhibiting factor was seen in the *lack of affinity and resentments*, especially among professional caregivers. This precondition was highlighted by interviewees from group 2 and 3:

“Professional nurses do not usually have a strong affinity for technology.” (Expert 15).

One expert saw the reason for this in the fact that “[ … ] *many people are still not clear about the role of technology and digitalization in relation to the profession and practical work*.” (Expert 12).

### 3.2 Politics - regulation - economy: who pays?

In the field “politics - regulation - economy,” *scarcity of public resources*, *growing private wealth*, *regulative advances* and *enhanced funding policy* were identified as accelerating the development and usage of IAT in dementia care. This field addresses the close interrelation between soft and hard law as well as political-economic aspects that impact the regulation of access, offers and demands.

The representative of a patient organization named the *scarcity of public resources*, especially in the nursing sector, as an accelerator for the implementation of new technologies:

“We have, of course, a scarcity of resources which could foster the use of such items” (Expert 1).

One technology expert differentiated this scarcity of resources regarding the dimensions of financial and human resources (Expert 18).

On the other hand, one expert from the healthcare administration of a federal state noted a general *growth of private wealth*. This makes it possible for private individuals to purchase modern assistive technologies:

“[ … ] on the demand side, it is also the growing prosperity that makes such things possible, so that I say: I’ll splurge on it [ … ].” (Expert 17).

Additionally, some experts stated that the demand for IAT is strengthened by *enhanced funding policies* for individual investments as well as for research projects. A participant from a private long-term care insurance stated:

“[ … ] today there are already funding programs via the KFW^1^ to subsidize the corresponding renovation work in private apartments [ … ]” (Expert 15).

In general, legal regulation in the area of digital healthcare “[ … ] *has picked up speed even more in recent years* [ … ]” (Expert 12), for example through the Digital Health Care Act (Expert 10; Expert 12). In addition, the federal government funds research in the area of assistive technologies (Expert 10).

Nevertheless, several inhibitors were likewise identified in this structural field: *unclear financing options*, an *uncluttered market*, *high business risks*, *data protection and ethical challenges*, as well as *too short research funding periods*.

In clear contrast to accelerating developments such as growing private wealth, experts form all groups problematized the as yet *unclear financing options* of IAT and TADC for private individuals. One technology researcher outlined the fundamental problem: “*If technology changes care, then this changed form of care must somehow pay for itself*” (Expert 2). Another technology researcher stated:

“It is, after all, always a question in care why many such interventions are not yet ready for the market or why they are not used, and then of course it would also be question of who ultimately bears the costs for expensive monitoring and assistive systems.” (Expert 6).

According to the experts, this has been insufficiently clarified. In this context, a representative of a welfare agency criticized restraints on the long-term care insurance providers:

“[ … ] that the insurance companies say, okay, we’ll do a pilot project [ … ], but on the other hand we are rather hesitant, for example, to expand the list of aids and to say that we think it’s good that something like this is used, and we’ll also finance it.” (Expert 5).

At the same time, private willingness to invest is too low in relation to growing private wealth, according to a representative of a long-term care insurance:

“One finding that we have taken away from our model program for the further development of new forms of housing is that, on the one hand, people are of course always grateful when apartment owner or housing cooperatives upgrade their apartments technically and digitally. The moment that this is then reflected in the rent and possibly associated with their own share, and perhaps with an increasing rent, then of course the willingness often drops.” (Expert 12).

The inhibiting effect of unclear financing may be reinforced by the cluttered market that some experts identified:

“It is not clear what is in the market, what is it actually good for.” (Expert 8)

This, in turn, would mean high business risks for technology companies (Expert 4; Expert 7).

Additionally, *legal requirements regarding data protection* were identified as a (time-)relevant hurdle for companies and insurances:

“Ensuring data protection and privacy for eighty million people is probably not an easy task” (Expert 12).

Legal requirements are an obstacle in this regard, especially in light of the quantity of data necessary for training algorithms (Expert 10). In addition, the application and approval procedures under the Medical Devices Act were seen as too extensive and time-consuming (Expert 8; Expert 10).

Moreover, *research funding periods* were criticized as *too short*. Due to the time limitations, the projects would often focus “[ … ] *technology development, technology design* [ … ]” (Expert 2) and would not include “[ … ] *technology use, technology appropriation, changes of structures, processes* [ … ]” (Ibid.). Hence, the research on the implementation of technologies are often neglected:

“[ … ] we actually lack this transfer and diffusion area, where you really look a lot in practice and also on what happens after the technology was implemented” (Ibid.).

### 3.3 Technology: using IAT in the digital desert?

In this field, the experts named general *advances in basic technical research* as well as the *enhanced customizability and interconnectivity* of technical devices as accelerating preconditions. “Technology” as a field includes material, methodological and scientific developments that determine advancements in technology developments.

Participants from all expert groups identified a variety of advances in basic technical research as particularly accelerating the development and use of IAT in dementia care:

“It starts with really very basic technological things, already starting with mechanical engineering up to AI technologies, especially in the field of image recognition, where it is about facial expression analysis, deep learning and so on [ … ].” (Expert 7).

These advances would enable new applications and open up further fields of use for IAT (Expert 10; Expert 4; Expert 17).

The *enhanced customizability and interconnectivity* of devices, which enables flexible use of artifacts, is particularly accelerating. One technology researcher cited smartphones as an example:

“So, if we just look at smartphones now, as a universal tool that I can adapt and expand as I want with apps, then we have an area here where we gain a lot of opportunities through the fact that technology becomes more networked, that it can be used more flexibly when I adapt it.” (Expert 2).

In the field of technology, the experts identified a *deficient digital infrastructure*, a *lack of potential users’ participation in technology development* and, thus, an *excessive focus on innovation*, as well as *dementia-specific challenges in development and implementation* and *challenges regarding data collection and data security* as inhibiting preconditions for IAT and TADC.

*Deficient digital infrastructure* was identified as a crucial inhibiting structural factor for IAT and TADC. For example, two participants from group 1 referred to a lack of “[ … ] *good and stable broadband network coverage* [ … ]” (Expert 7; Expert 20). This challenge comes to a head in the context of nursing homes, as a technology researcher highlighted:

“So, if we look at this digital gap, people who live in institutions [ … ] are very much cut off from internet coverage, for example; thus, they are disadvantaged there” (Expert 2).

This assessment was confirmed by a participant from a welfare agency and referred to the insights of the COVID19 pandemic:

“[ … ] the Corona pandemic also clearly showed that the nursing homes are not equipped with sufficient WLAN capacities [ … ]” (Expert 11).

The lack of interoperability, i.e. the uniformity of data and technologies, is another major obstacle, as Expert 5 stated: “[ … ] *in some places fax machines are still in use* [ … ].” One technology researcher was critical overall about the digital transformation in Germany, which “[ … ] *is so slowly and badly done* [ … ]” (Expert 13).

Moreover, the lack of potential users’ participation in technology development was stated as an inhibiting preconditions for IAT and TADC. This was especially highlighted regarding the group of professional caregivers:

“I don’t necessarily experience the willingness to do this in the field of nursing, which is understandable for me because what else am I supposed to do, now I’m also supposed to do ethics, now I’m also supposed to develop technologies [ … ].” (Expert 4).

According to other participants, the unequal distribution of digital literacy, which was stated as an inhibiting factor in the field of society, also plays a decisive role in this regard because professional nurses in particular lack the time in their everyday lives to acquire the necessary competencies (Expert 20). At the same time, the lack of participation leads to an *excessive focus on innovation* and often result in a lack of suitability of the products for everyday use (Expert 19; Expert 20).

Another inhibiting factor identified was *dementia-specific challenges in development and implementation* of new technologies. In particular, competence limitations associated with dementia-related syndromes were named as an obstacle to both the participation of those affected and the measurement of outcomes of technology-assisted interventions in dementia care: especially communication problems of persons with dementia, the progression and variance of dementia-related syndromes and doubtful or fluctuating capacity to consent (Expert 1; Expert 2; Expert 12).

Mirroring the legal requirements regarding data protection, which were mentioned in the field of politics - regulation - economy, experts identified *technical challenges in the area of data collection and data security* as inhibiting factors in the field of technology. The former, according to a technology researcher, requires to “[ … ] *inflicting pain on people in a controlled setting in order to then gain the necessary data* [ … ]” (Expert 10); this was described as both legally and ethically challenging and at the same time morally and psychologically stressful for the researchers (Ibid.; cf. Expert 13). In addition, there is a challenge of how to secure highly sensitive health-related data on a technical level: “*So, on the technical level* [ … ] *it is clearly the issue of how can I actually guarantee privacy and security of the data in such systems* [ … ]” (Expert 7); in this area, “[ … ] *there are currently dramatic shortcomings* [ … ]” (Ibid.).

## 4 Discussion

In order to empower people who have dementia, to relieve the burden on caregivers and to improve the quality of care, IAT are being used increasingly in dementia care. As we stated in the introduction, successful and empowering TADC depends to a significant degree on exogenous preconditions. These are social, political, economic, legal and technological factors which accelerate or inhibit access to TADC. German experts from technology research and development, healthcare policy and administration, long term care insurances and professional nursing assessed these preconditions as highly ambivalent. In the three different exogenous structural fields that we identified, the experts identified as the most crucial preconditions: societal digitalization, unequally distributed digital literacy, deficient digital infrastructure and unclear refinancing options for IAT.

In sum, the results of our study indicate that the development, implementation and use of IAT in dementia care takes place in a highly complex structural framework, which to a large extent preconfigures the success of socio-technical empowerment. Thus, structures matter more than ever. For this reason, we will formulate practical-ethical recommendations following the discussion. Thereby, we also contribute to a more structure-aware medical and caregiving ethics.

### 4.1 Practical-ethical considerations of TADC with regard to empowerment by considering fair access

In all three structural fields — society, politics - regulation - economy and technology—, the interviewees identified accelerating as well as inhibiting preconditions. Notably, the factors identified are not only highly ambivalent (some accelerating and inhibiting preconditions within one structural field contradict or even undermine each other). For example, digitalization vs. unequally distributed digital literacy, growing private wealth vs. unclear financing options or enhanced customizability and interconnectivity vs. deficient digital infrastructure. Moreover, inhibiting preconditions can, at worst, reinforce each other and, thus, counteract the accelerating factors. We thus assume that there is an intersectionality of inhibiting preconditions for TADC. Other authors have identified these tensional connections, too. Sowa et al., for instance, highlighted the function of socioeconomic status for healthy and successful ageing. Higher social status, income and education do not only influence lifestyle choices but also “[ … ] increase options for dealing with ill health by better opportunities for the health care use and quality of care” (27).

In the following, we discuss four preconditions in detail which we think are crucial for fair access to IAT, and thus for the very possibility of socio-technical empowerment. In consequence, an empowerment-ethical reflection —i.e., a reflection of preconditions for and impacts on (socio-technical) practices on independence, participation and self-determination— of TADC must consider the exogenous structures and preconditions which either accelerate or inhibit fair access to TADC and to related opportunities to a significant extent.

### 4.2 Digitalization

The interviewees identified digitalization, i.e. the penetration of all areas of life with digital technologies, as a social phenomenon. It is seen as one of the most important accelerating preconditions for the development, implementation and use of IAT in dementia care. This transformative trend is made possible and fostered especially by two characteristics of new technologies: pervasiveness and ubiquity (9). The new technologies are characterized as pervasive as they are available everywhere, for everyone, and at all times (ibid.; cf. 28, p. 293). They are ubiquitous as they are present in an ever increasing invisible, interconnected and non-intrusive way (9, 29, 30).

On the one hand, these characteristics accelerate the use of new technologies and enable their seamless integration into everyday life. Hence, digitalization could be interpreted both as a means and manifestation of fair access to new technologies and the regarding opportunities. On the other hand, the subtle penetration of everyday life with digital technologies entails social and ethical risks. For instance, questions arise as to whether denial of the technologies is still possible at all and whether (possible) denial is associated with social disadvantages, e.g. exclusion and new or exacerbated inequalities (31).

From an empowerment-ethical perspective, it is to be stated that digitalization is fundamentally linked to ethical challenges. On the one hand, it opens up new windows of opportunity for a more independent and self-determined life and enables ever more persons to use digital technologies. On the other hand, self-determination is undermined when there is no possibility to opt-out, and social participation is challenged when it is no longer possible to engage in social life in a non-digital way.

### 4.3 Unequally distributed digital literacy

The digitalization is undermined by several other preconditions. One of the most influential of these is unequally distributed digital literacy. As Sowa and colleagues highlight, the educational status of a person exerts considerable impact on healthy and successful ageing (27). Furthermore, education is a crucial element of empowerment practices (32–34). Education enables persons to critical thinking and realization of their own interests (35–37). Thus, digital education and digital literacy is key for autonomous decisions regarding the use of IAT. Against this background, digital literacy can be defined in two ways: first, as the sum of competencies necessary to use digital technologies in a proper and critically reflected manner (38, 39); second and relatedly, as the (informal) educational status regarding digitalization in general and digital devices in particular.

In general, various interviewees described this status as very low in the general German population. In particular, some experts problematized a lack of digital literacy among professional caregivers which has also been considered by other authors (40–42). Furthermore, an expert from the field of technology development mentioned the fact that some older people and people in need for care have younger, digital literate relatives and some not; this means that the latter group has no opportunity to acquire digital competencies in a low-threshold and informal way, thus being disadvantaged in comparison to the first group. This challenge to fair access is even exacerbated by the before mentioned lack of digital literacy among professional caregivers as these are the only source of information about IAT and TADC for many older persons without young, digital literate relatives.

From an empowerment-ethical perspective, the unequal distribution of digital literacy must, hence, be characterized as a major obstacle to the self-determined use of IAT and to fair access to TADC in at least three dimensions. Firstly, this inequality is a symptom of unequal access to competencies. Second, lacking necessary digital literacy challenge the self-determination of persons in need for care as they cannot assess adequately the opportunities and risks related to IAT. Finally, a lack of digital literacy limits fair access to TADC: When not knowing about existing —and potentially useful— devices and systems, one cannot participate in their implementation and usage.

### 4.4 Deficient digital infrastructure

Another crucial inhibiting precondition is the deficient digital infrastructure in Germany. This refers in particular to lacking broadband internet coverages in rural areas and lacking access to WLAN in nursing institutions.

This challenge, is, however, not only prevalent in Germany but also in other countries. For instance, Vollmer Dahlke and Ort (43) have noted that 24 million US citizens are currently living in so-called digital deserts, i.e. areas without access to broadband internet. This access is, however, “[ … ] a prerequisite to telemedicine use” (44). Accordingly, Loccoh et al (40) identified a correlation of health care access and internet service availability in the United States: “health care deserts” —ie., areas with “[ … ] poor access to domains of pharmacies, hospital, hospital beds, trauma centers, primary care physicians, and low-cost health centers [ … ]” (ibid., p. 1)— are often simultaneously digital deserts. They conclude that, when not accompanied by efforts to improve internet access, “[ … ] telemedicine expansions may have low effectiveness in counties where telemedicine is most needed [ … ]” (ibid., p. 2). On the contrary, most probably such expansions would even reinforce rural-urban health disparities and the digital divide (9, 45).

It should be noted that deficient digital infrastructure fundamentally counteracts the trend of digitalization: Especially the disparities between rural and urban areas regarding fast and stable internet cause unequal opportunities to the usage of IAT and TADC. Even worse, already existing inequalities could even be reinforced. With a special focus on dementia care, this finding becomes even more tragic: Compared to metropolitan areas, the rural areas of Germany are not only digitally and health care-related disadvantaged but they also face the highest percentage of ageing persons and persons affected by dementia. Hence, these areas would benefit the most by TADC but are more and ever more disadvantaged due to lacking digital infrastructure.

### 4.5 Unclear and missing financing options

Alongside with digital illiteracy and deficient digital infrastructure, the interviewed experts identified unclear and missing financing options for IAT and TADC. As most IAT are or will be quite expensive (9, 46, 47), they stated that the acquisition is a challenge for both private users as well as institutional care facilities. Also internationally, the affordability and costs of IAT are considered in the discourse on IAT and TADC. The socioeconomic status is identified as a relevant exogenous precondition for the use of IAT and is linked with issues of fairness regarding persons with dementia and their relatives (9, 31, 48, 49). In several empirical studies on the implementation of assistive technologies, costs were identified as a major criterium of acceptability (48, 50). Accordingly, the most common reasons for persons with dementia and their caregivers not to use IAT was the high cost and the nonexistent refunding possibilities (7, 49). Remarkably, none of these studies cited concrete numbers that would prove the high costs.

Conversely, in our interview study experts from private insurances as well as from publicly funded care services and one from a welfare agency criticized private individuals for their unwillingness to invest private money in IAT. They stated, that for example people’s willingness to purchase smart home systems themselves or to contribute to their implementation by paying higher rents is very low. In sum, it is not yet clarified who shall and who can bear the costs for IAT and TADC neither whether and to what extent public co-funding should be implemented.

Unclear and missing financing options bear the risks of exacerbating already existing disparities regarding long term care supply between upper and middle-to-lower class seniors (49). Middle-to-lower economic status of individuals is an (at least) threefold inhibitor for using IAT: first regarding the purchase for acquisition, second regarding maintenance-related costs and third, on an even more fundamental level, the socio-economic status of regions has significant impact on the supply with internet access. Vollmer Dahlke and Ort (43) elucidate that commercial internet providers try to avoid the economic risk of equipping rural areas with broadband internet associated with the probably non-usage by residents due to high costs.

With regard to this economic factor, the before mentioned intersectionality of exogenous preconditions becomes most evident: Individuals with restrained financial resources —regarding their own economic status and that of their relatives— are not free to decide whether or not to use IAT or participate in TADC and, thus are violated in their self-determination. Participation in the opportunities of TADC, hence, becomes a matter of economic class and not of evident-based need assessment.

## 5 Conclusions and policy recommendations

If TADC should be established as a socio-technical practice of empowerment for persons with dementia, the identified and discussed preconditions must not stay out of the focus of ethics and health policies. Health literacy as well as digital literacy, access to internet, and refinancing-based independence from the socioeconomic status are prerequisites for fair access to TADC.

In order to inform the scientific community and other relevant stakeholders and to equip them with normatively founded orientation, we propose some healthcare policy-related practical-ethical recommendations. Even if our discursive context is Germany with its special health care system, we try to formulate the recommendations in a more generalized manner as we think they are relevant for all high-industrialized, high-income and democratic contexts.

1. Digitalization seems to be a global transformative trend which entails many opportunities, but it has to be shaped by the society in the most participatory, democratic manner. The pervasiveness and ubiquity of new AI-based technologies and related autonomous systems must not undermine the self-determination of patients. It is important to ensure each single individual’s choice whether or not to use these technologies or to participate digitally in social life. In order to guarantee this possibility of free choice, health care and long-term care policy should guarantee that existing human-human contact and opportunities to analogue social interaction are not being replaced by technology. The normative goal of fair access has to entail the possibility to veto or deny TADC on a case-by-case decision.
2. Residents of rural areas are often living in “digital deserts.” They do not have equal access to digital technologies in comparison to residents of urban areas. Hence, relevant policy makers must maintain and strengthen initiatives to equip rural areas with broadband internet in order to establish fair access to modern health care.
3. With regard to residents of long-term care facilities, private and social welfare providers are called upon to apply adequate access to internet for their residents. The financing has to be solved efficiently and in favor of the yet disadvantaged residents. This must comprise public-private partnerships to ensure affordable access to stable and fast internet in the facilities. As this access is a fundamental precondition for modern health care, it has to be prioritized by policy makers.
4. The normative ideal of good ageing remains valid across the entire continuum of care and regardless of housing arrangements. In contrast, those living in rental properties often cannot decide autonomously about constructional adaptions to barrier-free living. They are dependent on the owners’ consent for such measures. Therefore, relevant stakeholders and decision-makers in politics and long-term care insurances should consider facilitating the constructional adaption for the purpose of IAT-assisted living. Furthermore, public housing subsidies should generally accelerate the creation of barrier-free, IAT-adaptable residential units.
5. Digital literacy is particularly important for enabling the self-determined use of IAT and for achieving the goals of TADC. Therefore, various initiatives need to be taken to increase the digital literacy of persons with dementia, their relatives and professional caregivers. In our opinion, the necessary digital skills that need to be acquired include: knowledge about available devices and systems; knowledge about the costs of IAT and TADC; knowledge about the opportunities and risks associated with the usage of IAT; basic knowledge about the sort and amount of health data collected and processed; the ability to use the IAT devices properly; knowledge about how to withdraw consent in (aspects of) data processing in any phase of use; and knowledge about how to shut down a device as ultimate measure of self-determination. As digital literacy is key to self-determined, i.e. well informed and critically reflected, decisions about whether or not to use IAT, digital education for health contexts should be provided not only in old age, but already in school. With regard to the present older generation, educational measures and offers have to be installed in order to equip older persons with the necessary level of digital literacy. These offers have to be low-threshold, on site, and free or low-cost, e.g. in community colleges. In order to strengthen the users’ capacity for digital decision-making in TADC, methods should be developed to visualize the sort and amount of collected and processed data during the entire use of IAT.
6. With regard to the lack of digital literacy among the group of professional caregivers, the professionals themselves as well as the policy makers need to be addressed: digital literacy and competencies must be recognized a part of healthcare professionalism. Professional associations of healthcare and long-term care personnel should promote this change in professionalism by supporting their members in acquiring necessary competencies by mandatory courses in professional trainings.

## Data Availability

Due to the data protection agreement with the participants and our ethics approval by University Center Göttingen, we cannot provide the raw data. Requests to access the datasets should be directed to the corresponding author.

## Footnotes

1 KFW: Kreditanstalt für Wiederaufbau, is a German state-owned investment and development bank (https://kfw.de/kfw.de.html).
